# *In Silico* Generation of Peptides by Replica Exchange Monte Carlo: Docking-Based Optimization of Maltose-Binding-Protein Ligands

**DOI:** 10.1371/journal.pone.0133571

**Published:** 2015-08-07

**Authors:** Anna Russo, Pasqualina Liana Scognamiglio, Rolando Pablo Hong Enriquez, Carlo Santambrogio, Rita Grandori, Daniela Marasco, Antonio Giordano, Giacinto Scoles, Sara Fortuna

**Affiliations:** 1 Department of Medical and Biological Sciences, University of Udine, Piazzale Kolbe, Udine, Italy; 2 Department of Medical Biotechnology, University of Siena, Policlinico Le Scotte, Viale Bracci, Siena, Italy; 3 Department of Pharmacy, CIRPEB: Centro Interuniversitario di Ricerca sui Peptidi Bioattivi- University of Naples “Federico II”, DFM-Scarl, Naples, Italy; 4 Department of Drug Discovery and Development, Italian Institute of Technology (IIT), Genova, Italy; 5 Department of Biotechnology and Biosciences, University of Milano-Bicocca, Piazza della Scienza, Milan, Italy; 6 Sbarro Institute for Cancer Research and Molecular Medicine & Center for Biotechnology Temple University Philadelphia, Pennsylvania, United States of America; 7 Department of Medicine, Surgery & Neuroscience University of Siena, Strada delle Scotte n. 6, Siena, Italy; 8 Department of Biology, Temple University, Philadelphia, Pennsylvania, United States of America; University of Michigan, UNITED STATES

## Abstract

Short peptides can be designed *in silico* and synthesized through automated techniques, making them advantageous and versatile protein binders. A number of docking-based algorithms allow for a computational screening of peptides as binders. Here we developed *ex-novo* peptides targeting the maltose site of the Maltose Binding Protein, the prototypical system for the study of protein ligand recognition. We used a Monte Carlo based protocol, to computationally evolve a set of octapeptides starting from a polialanine sequence. We screened *in silico* the candidate peptides and characterized their binding abilities by surface plasmon resonance, fluorescence and electrospray ionization mass spectrometry assays. These experiments showed the designed binders to recognize their target with micromolar affinity. We finally discuss the obtained results in the light of further improvement in the *ex-novo* optimization of peptide based binders.

## Introduction

The design of new ligands and receptors for protein recognition is a key step towards the development of new diagnostic tools and selective drugs. So far antibodies represent the biomolecules with the highest affinity and selectivity toward proteins [1,2,3]. Notably, they are usually employed in a number of diagnostic applications such as ELISA and immunohistochemical assays. However both their production and optimization are expensive and time consuming requiring either *in vitro* (cell line culture and monoclonal screening) or *in vivo* procedures (animal immunization) [4]. Peptides are an emerging alternative to antibodies as drug candidates [5], anti-aggregation agents [6], and probes for molecular recognition [7]. They can be automatically synthesized offering a wide variety of chemical targeted modifications, such as fluorescent or affinity tags. They are highly versatile while composed by a limited number of building blocks and their sequences are typically extracted from protein-binding peptides or protein domains [8,9] or optimized by phage-display libraries [10,11,12] sometimes reaching picomolar affinity towards their targets [12].

Given a particular peptide sequence targeting a protein, and when its binding site is known, a number of docking algorithms can provide an accurate structural model of the complex[13]: FlexPepDock [14,15], PepCrawler [16], HADDOCK [17], AutoDock Vina [18], or GAsDock [19]. These are generally capable of suggesting possible binders out of a number of possibilities. However, while performing accurately in their structural determination task, these algorithms are generally not capable of accurately estimating the dissociation constants between peptides and proteins. While successful attempts in this direction have been made [20,21,22], plain docking disregarding this issue has been successfully embedded into a number of algorithms for the *ex-novo* optimization of short peptides allowing random sequences to evolve towards a high affinity to their target.

Typical molecular optimization algorithms embedded in evolutionary codes are found in the form of genetic algorithms [23,24] (allowing a population of molecules to be scored, selected, mixed, and mutated with the goal of maximizing their score), or Monte Carlo (MC) based algorithms such as simulated annealing [25,26,27]], and replica exchange Monte Carlo (REMC) where the system is allowed to explore high temperature unfavorable configurations to escape local minima [28], or agent-based algorithms where molecules can follow arbitrary sets of rules to reach a low score [29,30]. These algorithms have been successfully used to optimize clusters [23,27,30], molecular conformations [24,25], protein conformations [28], supramolecular structures [29]. In all cases the protocols are designed to minimize the system “score” generally corresponding to the system or the interaction energy between its components.

When optimizing peptides as binders the score is generally the output of an existing docking code [31] and these have been embedded in protocols capable of evolving random sequences. For instance MOE Dock, a docking algorithm based on Monte Carlo (MC) simulated annealing, has been incorporated in a genetic algorithm to generate tetrapeptides with dissociation constant K_D_ = 60μM with quino-protein glucose dehydrogenase [32] and also to generate α-synuclein aggregation modulators with K_D_ = 19μM [33]. AutoDock Vina has been implemented in ENDPA, another genetic-algorithm-based code, and used for generating propyl oligopeptidase, p53 and DNA gyrase ligands[34]. The Leap-Frog genetic algorithm based search engine together with the commercial FlexiDock has been used to generate octamers and 13-mers with micromolar affinity for Ochratoxin A[35]. Higher affinity has been reached by an algorithm based on a combination of molecular dynamics, semiflexible docking using Autodock Vina, and replica exchange Monte Carlo (REMC[36]). Using this protocol a deca-alanine was shown to evolve to a final sequence showing nanomolar affinity towards the antiretroviral drug efavirenz[37].

**Fig 1:**
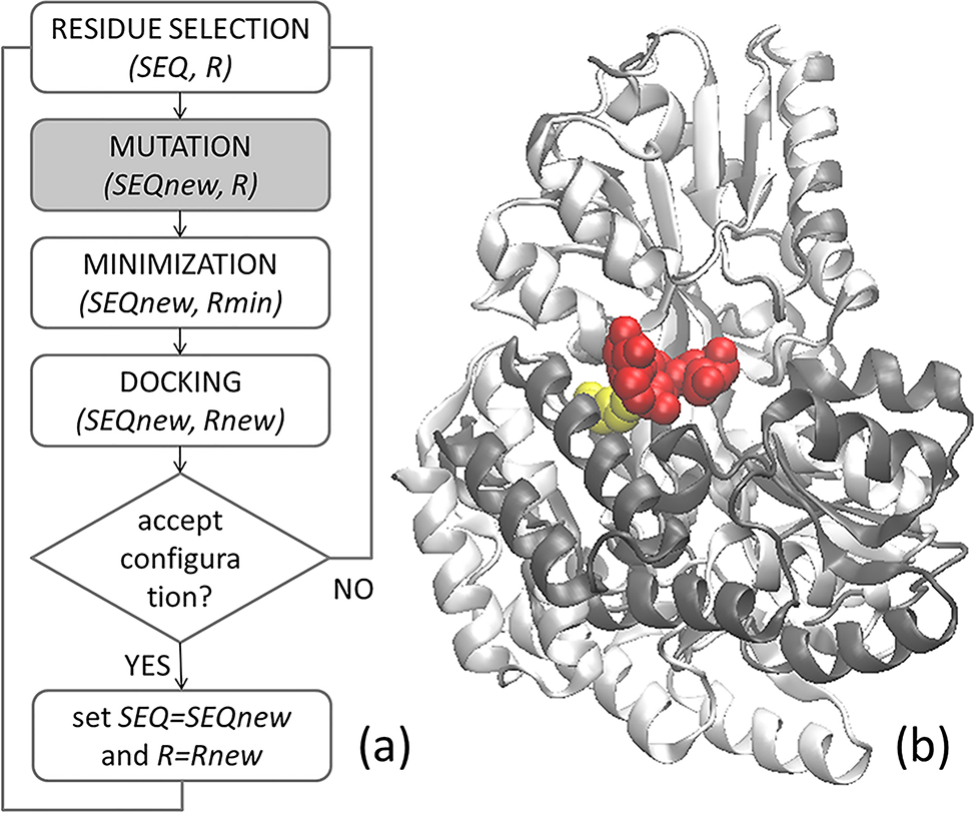
(a) Flowchart of the peptide-optimization algorithm. (b) Structure of MBP in open (white, PBD code 1OMP) and closed (grey, PBD code 3MBP) configurations in which the coordinated maltotriose (red) and the Met330 (yellow) are highlighted.

Here we aim to evolve *in silico* protein-binding octapeptides starting from a random sequence. We use the Vina-based approach of Ref.[37] (descibed in Fig 1A), that successfully generated drug recognizing peptides of nanomolar affinity, to generate a number of protein binding peptides. We chose the maltose binding protein (MBP, Fig 1B) as a test case and a set of peptides computationally designed to bind to its maltose binding site. MBP is the prototypical member [38] of the periplasmic binding proteins also called “gold mine for the study of protein-ligand recognition” [39]. MBP, with a molecular mass of 42 kDa, is an ellipsoidal monomeric protein consisting of two globular domains linked by a flexible multistranded region. The MBP is known to exist in two conformations: a populated open state (95%) and a closed state with the two globular domains approaching each other [40]. The flexible region defines a binding pocket for maltose, maltotriose, and other maltodextrins [41,42]. Upon binding to its native ligands, the MBP conformational equilibrium shifts towards the closed form, a behavior shared by all periplasmic binding proteins [43].

The paper is organized as follows: (i) in Sec.2.1 the peptide:MBP complexes are computationally generated following the protocol schematized in Fig 1A and screened by means of an empirical docking-based method to identify the binder with higher affinity towards a defined protein binding site; (ii) the peptide:MBP complex stoichiometry is then determined in Sec.2.2 through Electrospray Ionization Mass Spectrometry (ESI-MS) and in Sec.2.3 the binding affinity is experimentally determined by means of Surface Plasmon Resonance (SPR) and Fluorescence Spectroscopy; (iii) in Sec.2.4, by comparison of experimental affinities and theoretical predictions, we demonstrate the computationally generated peptides to converge towards a consensus sequence; in Sec.3, we discuss the results with the goal of pinpointing possible strategies for the development of accurate computational protocols for peptide design.

## Results

### 2.1 Computational generation and characterization of peptide ligands

The algorithm, schematized in Fig 1A, was designed to optimize sequence (SEQ) and conformation (*R*) of a random starting peptide of fixed length. Its kernel iteratively (i) mutates the primary structure of the peptide, (ii) relaxes the newly mutated structure, and (iii) docks the new structure to the target assigning it a docking score. All the procedure is carried out in vacuum. In the last step (iv) the mutation is accepted or rejected following a MC acceptance probability:

$$P_{\text{MC}}=\text{min}\left[1,\text{exp}\left({\text{S}}_{\text{new}}-{\text{S}}_{\text{old}}\right)/{\text{k}}_{\text{B}}\text{T}\right]$$

where S_old_ is the docking score of the starting peptide, S_new_ is the docking score of the mutated peptide, k_B_T a tunable parameter which defines the acceptance probability of unfavorable mutations. The algorithm runs in parallel at three different k_B_T. At every step a REMC swap is attempted between two configurations belonging to two randomly selected k_B_T. The three different temperatures guaranteed appropriate mixing of the system allowing the exploration of high energy configurations while pushing the system towards lower energy configurations.

**Fig 2:**
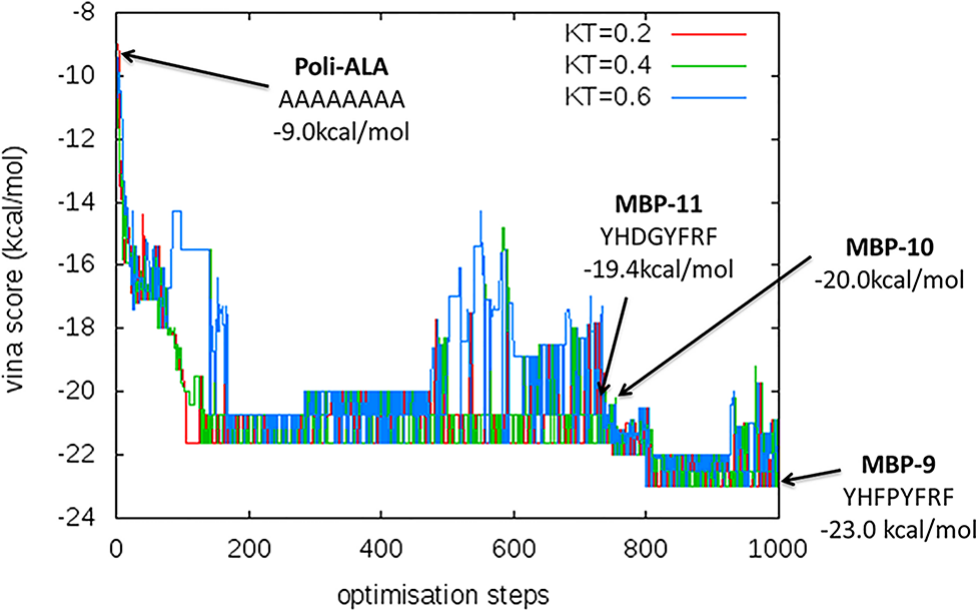
Computational generation of peptides. Autodock Vina binding energy evolution as a function of the optimization steps for the generation of MBP-9. Arrows indicate the energy associated with the starting poly-alanine, MBP-9, and two peptides along the minimization path: MBP-10 and MBP-11.

We performed the molecular design on the crystal structure of the MBP in its open configuration (in white in Fig 1B) and we selected the region surrounding Met330 (in yellow in Fig 1B) as the binding site. We optimized a set of octapeptides by running the optimization algorithm for 100 steps at k_B_T = 0.2, 0.4, 0.6 using in all cases a linear octa-alanine as the starting sequence, following the protocol of Ref. [37]. We ran the optimization code 9 times, selecting the lowest scoring end-simulation peptide of each run for further analysis. Scores are Autodock Vina estimated binding affinities in kcal/mol. The binding affinity evolution observed in a typical run is shown in Fig 2. From the run of Fig 2, we further selected two peptides along the minimization path and the starting poly-alanine. Moreover, due to the low aqueous solubility of the poly-alanine, we also considered the sequence AAARRAAA as a negative control. Overall, for further screening, we chose the 13 peptides reported in Table 1.

**Table 1:** Average docking scores. Docking scores are calculated with Autodock Vina over 10 runs each generating 9 configurations, and with MC+Vina over the last 10 configurations of 10 runs. Errors are standard deviations over all the samples. In parentheses the protein configuration used for the docking. All the values are in kcal/mol.

Peptide	sequence	Vina (1OMP)	MC+Vina (1OMP)	MC+Vina (3MBP)
**MBP-1**	SPAGGQDF	-7.8±0.6	-14.7±1.6	-11.8±0.9
**MBP-2**	WGTNGGTR	-7.7±0.5	-14.1±1.4	-12.1±1.3
**MBP-3**	APRGGNTS	-7.0±0.6	-13.8±1.2	-11.5±0.6
**MBP-4**	PQYPPHDN	-8.0±0.6	-15.1±1.0	-11.1±1.3
**MBP-5**	GLPKPGGN	-6.6±0.3	-12.5±0.5	-10.3±0.7
**MBP-6**	PQKGGMWD	-7.1±0.4	-14.9±1.3	-10.7±0.9
**MBP-7**	WSPNFWWR	-8.3±0.6	-16.8±1.5	-11.9±1.5
**MBP-8**	WHPRPVWE	-8.3±0.3	-14.8±1.1	-10.1±1.7
**MBP-9**	YHFPYFRF	-8.9±0.7	-18.7±1.1	-10.5±2.5
***MBP-10***	*YGDGYFRF*	*-8*.*1±0*.*4*	*-15*.*1±1*.*4*	-12.6±2.0
***MBP-11***	*YHDGYFRF*	*-8*.*1±0*.*5*	*-16*.*2±2*.*4*	-11.8±1.7
***poliALA***	*AAAAAAAA*	*-7*.*1±0*.*3*	*-11*.*0±1*.*0*	-9.3±0.5
***NEG***	*AAARRAAA*	*-6*.*9±0*.*4*	*-13*.*3±1*.*1*	-11.5±1.6

**Fig 3:**
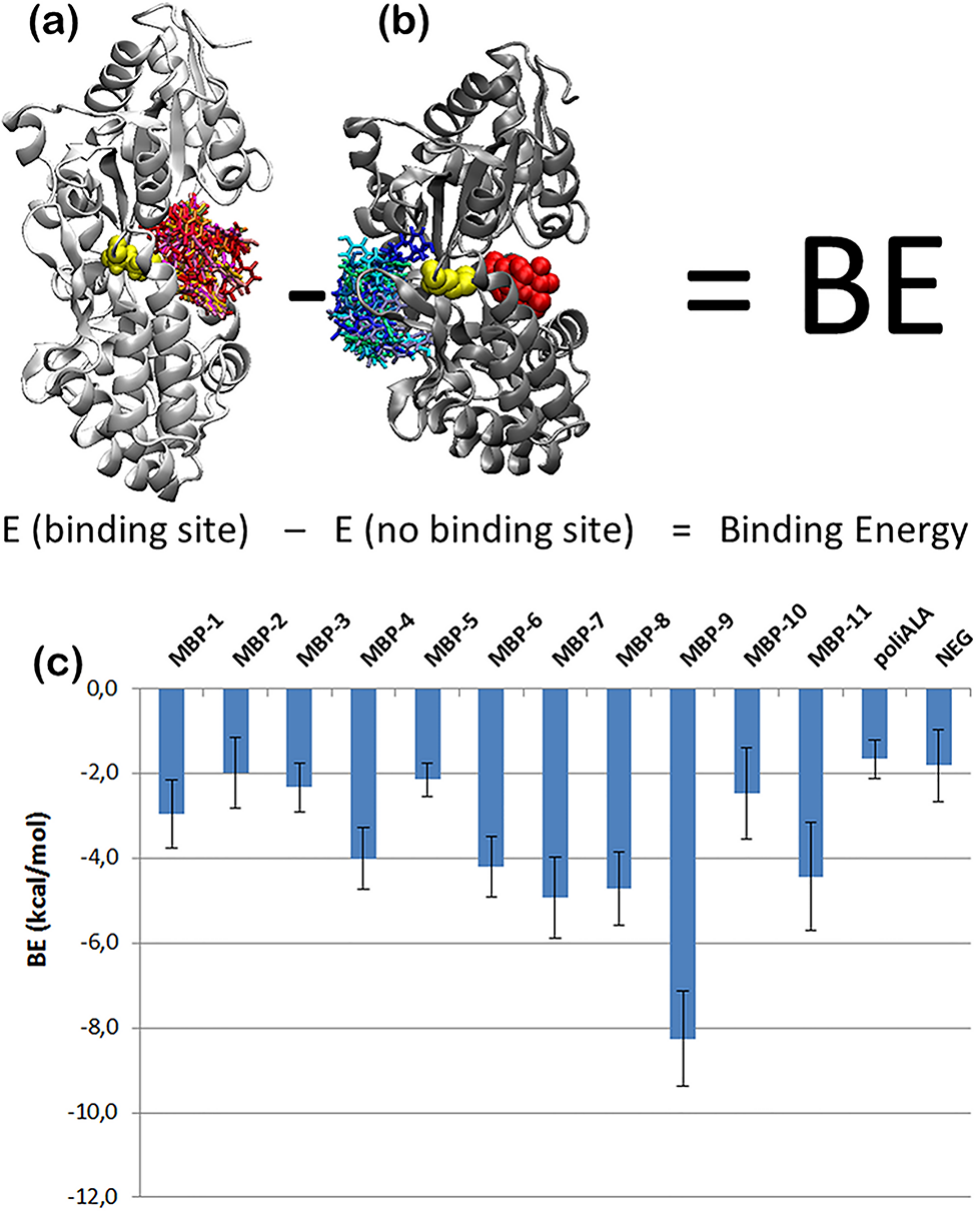
(a-b) Overlap of the 10 peptides final configurations obtained by redocking 10 times MBP-9 with the Vina+MC code for 100 steps at kBT = 0.6 on the Met330 site of the MBP (yellow) in open conformation (a) and in its maltotriose (red) containing closed conformation (b). (c) The MC+Vina calculated BE where the errorbars are the sums of the standard errors of the means calculated over the last 10 configurations of 10 runs of the docking protocols.

To characterize the chosen peptides we performed multiple dockings by switching off the mutation step of the optimization code (highlighted in grey in Fig 1A) and redocked all the 13 peptides at k_B_T = 0.6 (MC+Vina scores in Table 1). For comparison, we docked the same peptides also with the original Autodock (Vina scores in Table 1). Due to the stochastic character of the methods the end configurations widely varied but all were correctly located inside the pocket (as shown in Fig 3A and Figure A in **S1 File**). Accordingly the standard deviation on their docking scores was of 5% for MBP-9 and up to the 15% for MBP-11. In general, the inclusion of MC steps in Vina greatly improved the chances of finding a low energy configuration: the Vina scores were between -7.5 and -10.2kcal/mol, while MC+Vina found configurations with binding affinities between -12.3 and -20.2 kcal/mol, almost twice the binding affinities of the configurations found by the original Vina. By redocking the peptides both with Vina and MC+Vina we did not obtain the same binding energies we obtained for their generation (indicated by arrows Fig 2). Further, MC+Vina identified the poly-ALA as the peptide with the least favorable binding energy. Similar results were obtained by docking all the peptide with the same REMC protocol used for their generation. We further validated our results by refining the MC+Vina lowest energy configurations with FlexPepDock (see Table A and Figure A in **S1 File**).

The preliminary experimental results for the first eight peptides of Table 1 pointed toward a high micromolar affinity (3.28–5.11 kcal/mol at room temperature according to the relation ΔG = RTln(k_D_) where ΔG is the binding affinity R the ideal gas constant, T the system temperature, and the k_D_ measures are those collected in Table 2 and discussed in Sec.2.4). Since the result is well below the theoretically estimated binding affinity, we assumed that the abnormally high affinity values were due to the docking algorithm missing a number of sequence dependent entropic contributions. In order to correct these errors, we observed that the calculated energy was nonzero when docking a peptide to a non-binding site (as in the fifth column of Table 1). Thus we assumed this latter energy should be balanced by missing sequence dependent terms and applied a correction to the binding energy, defining the corrected binding energy as:

$$\text{BE}=<\;{\text{E}}_{\text{binding site}}\;>-<\;{\text{E}}_{\text{no binding site}}\;>$$

where <E_binding site_> is the average interaction energy of a peptide docked to its binding site, while <E_no binding site_> is the average interaction energy of the same peptide docked to a non-binding site. We employed the MC+Vina scoring protocol able to find the lowest energy configurations for the peptides of Table 1. We ran multiple dockings generating a number of possible MBP:peptide complexes targeting the maltose binding site in the open protein configuration (Fig 3A) and in closed maltose containing configuration (Fig 3B) at k_B_T = 0.6. While it is not possible to exclude a priori the aspecific binding of the peptides to either of the globular domains, we assumed no binding was possible in the maltose containing closed configuration, an assumption confirmed by ESI-MS experiments (see Sec.2.2 and Sec.2.4). We averaged the energies over the last 10 configurations of 10 runs of the docking protocols (last two columns of Table 1). The difference between the two energies for each peptide is plotted in Fig 3C showing that negative controls have the lowest BE among the peptides, and the other binding energies are in the micromolar range. Now MBP-9 emerges as a clear outlier and the best candidate for binding to the MBP.

**Table 2:** k_D_ (SPR determined) and pI values for the MBP peptides. CBE (Cannot be Estimated) is related to experiments in which signal variations were observed but K_D_ estimation cannot be reached neither through kinetic nor saturation analysis.

Peptide	Sequence	K_D_ (μM)	pI
MBP-1	**SPAGGQDF**	**4200 ±1400**	**3.80**
MBP-2	**WGTNGGTR**	**CBE**	**9.75**
MBP-3	**APRGGNTS**	**1500 ±200**	**9.79**
MBP-4	**PQYPPHDN**	**CBE**	**5.08**
MBP-5	**GLPKPGGN**	**CBE**	**8.75**
MBP-6	**PQKGGMWD**	**1300 ± 500**	**6.26**
MBP-7	**WSPNFWWR**	**CBE**	**9.75**
MBP-8	**WHPRPVWE**	**200 ± 5**	**6.75**
MBP-9	**YHFPYFRF**	**72 ± 3**	**8.60**
MBP-10	**YGDGYFRF**	**No binding**	**5.83**
MBP-11	**YHDGYFRF**	**No binding**	**6.74**

### 2.2 Stoichiometry of peptide:protein complex through ESI-MS

**Fig 4:**
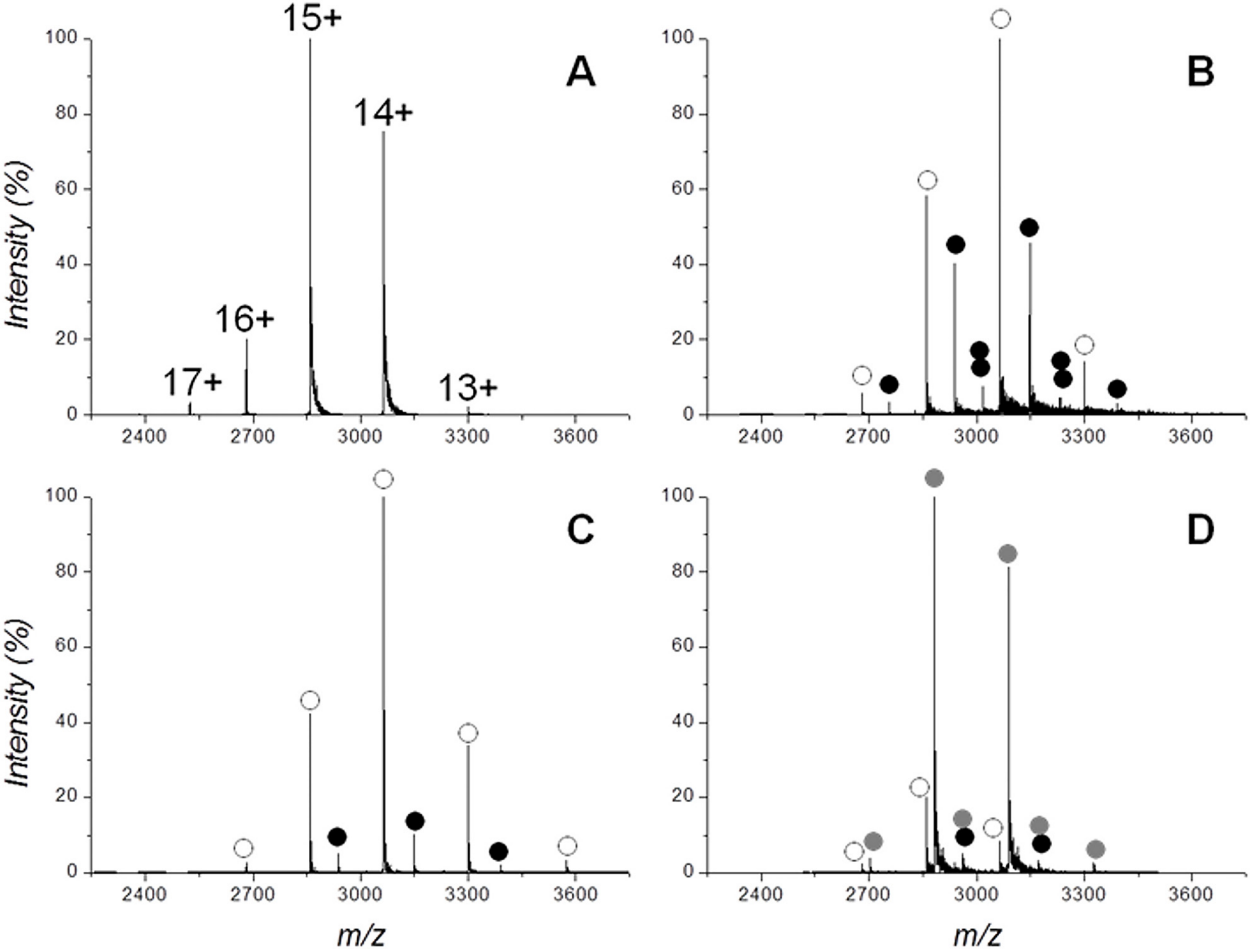
ESI-MS analysis of Protein-Peptide complexes. Spectra of 20 μM MBP in 50 mM ammonium acetate pH7. (A) DP60 V, (B) DP 60 V in the presence of 20 μM MBP-9, (C) DP 180 V in the presence of 20 μM MBP-9, (D) DP 60 V in the presence of 20 μM MBP-9 and 20 μM maltose. In panel A the peaks are labeled by the charge acquired during the electrospray. In panels B-D the peaks corresponding to apoMBP (○), MBP:MBP-9 1:1 complex (•), MBP:MBP-9 1:2 complex (••), MBP:maltose 1:1 complex (gray solid circle) and MBP:MBP-9:maltose: 1:1:1 complex (black/gray solid circles) are labeled.

The binding between the sequence endowed with the highest putative affinity toward MBP, namely MBP-9, was analyzed using the ESI-MS technique (Fig 4). The protein alone gave a narrow charge-states distribution centered on the peaks corresponding to the 14+/15+ charge states (Fig 4A), suggesting a compact and structured conformation of the protein.

In the presence of an equimolar amount of MBP-9 new peaks arose in the spectrum (Fig 4B), corresponding to the MBP:MBP-9 complexes with a 1:1 and 1:2 protein:peptide stoichiometries. The protein in the 1:1 and 1:2 bound states were ~33% and ~5% of the total amount, respectively. Protein:peptide complexes can be almost completely dissociated in the gas-phase by increasing the declustering potential (DP) of the spectrometer from 60 to 180 V (Fig 4C). As a negative control, we choose two standard proteins with a molecular mass similar to MBP: Ovalbumin (42.7 kDa) and Neuroserpin (46.3 kDa). The mass spectra of these proteins in the presence of MBP-9 were acquired under the same experimental conditions, and no peaks corresponding to protein:peptide complexes were detected (data not shown), indicating a specific recognition of MBP-9 toward the MBP.

Competitive experiments between MBP-9 and maltose were also performed through ESI-MS analyses, employing an equimolar mixture of MBP, MBP-9, and maltose (Fig 4D). Under these conditions, the great majority of the protein was bound exclusively to maltose, in agreement with its higher affinity (∼1μM)[44]. Only a small amount (less than 5%) can be referred to MBP bound to both ligands (MBP-9 and maltose) (Fig 4B). Therefore, the occupation of MBP pocket by maltose almost completely inhibits the binding of MBP-9, confirming that confirming that designed peptide binds in the same site of the sugar.

### 2.3 Estimation of dissociation constants of the peptide:protein complexes

**Fig 5:**
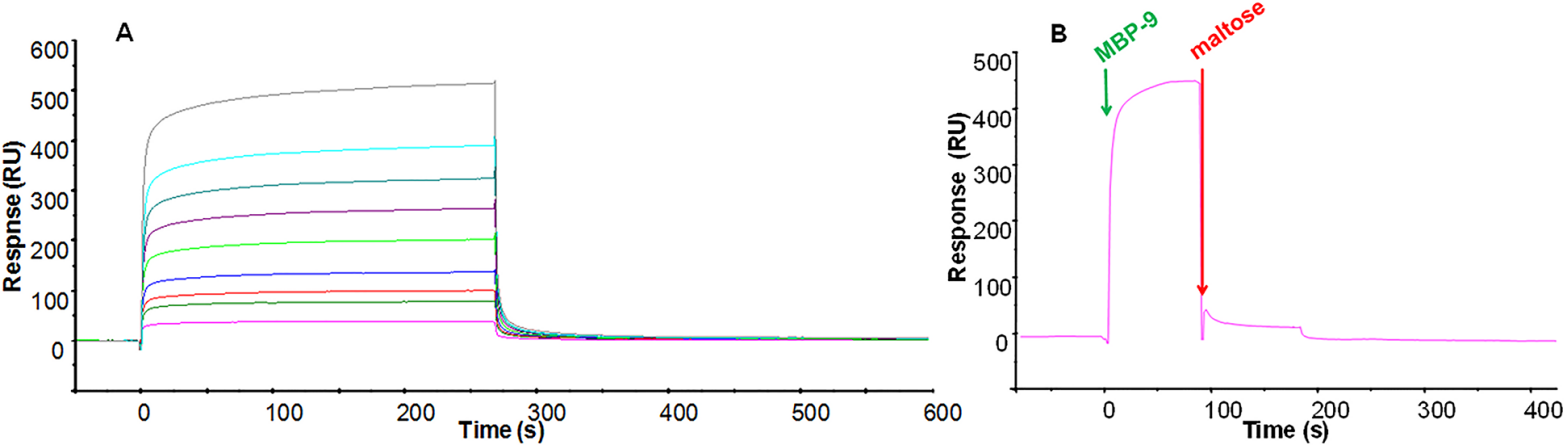
Overlay of SPR sensorgrams for the interaction between the immobilized MBP protein and MBP-9 (A). The experimental curves corresponding to different concentrations of peptides (0–800μM) were fitted according to a single binding model with 1:1 stoichiometry. Competitive assay between peptides and maltose for immobilized MBP: Sensorgram of the co-injection of MBP-9 peptide at 200μM and maltose at 20mM (B).

SPR was employed to evaluate the affinities of designed peptides towards the MBP protein. SPR experiments were carried out by immobilizing MBP on a CM5 chip with an immobilization level of 4900 RU, while peptides were employed as analytes. The analysis of MBP-9 experiments provided dissociation constants in the micromolar range K_D_ = 72±3 μM, by applying a 1:1 Langmuir model (Fig 5A).

**Fig 6:**
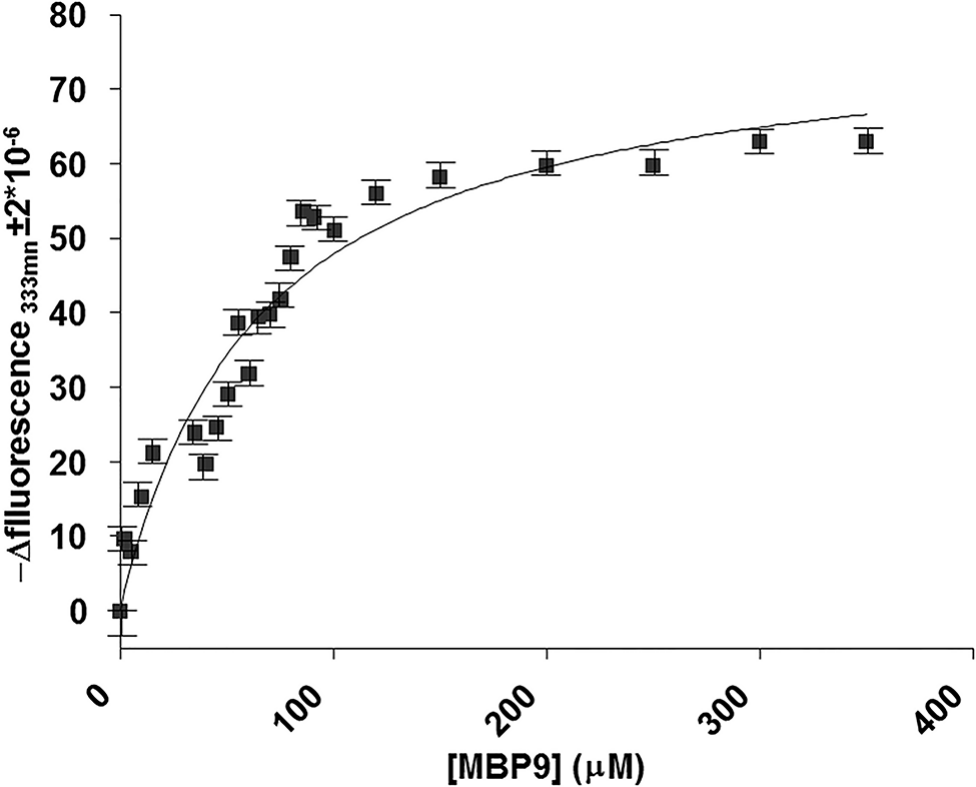
Tryptophan fluorescence quenching analysis showing the dose-response curve of the fluorescence values of MBP protein at 333nm plotted against the concentration values of MPB-9.

To assess the specificity of the recognition mechanism of the designed peptides we carried out competitive binding experiments through co-injection of peptides and maltose. MBP-9 at 200μM was co-injected with maltose in a 1:100 ratio. The recorded sensogram (Fig 5B) showed a dramatically decrease of the RU signal upon the presence of maltose. In this experiment the peptide concentration was twofold greater than K_D_, thus MBP protein was nearly saturated and consequently the signal decrease resulted from peptide displacement. Further, to corroborate the affinity values evaluated through SPR using an in-solution binding assay, we performed fluorescence experiments on the MBP-9:protein complex. Tryptophan fluorescence emission at 333 nm showed a dose-response quenching upon MBP-9 addition and -Δ fluorescence intensity was plotted against the concentration values of MPB-9 (Fig 6). Data were fitted with a 1:1 model of interaction, providing a K_D_ = 65 ±10μM, in agreement with the SPR measurement.

### 2.4 Comparison between theoretical and experimental binding affinities

The SPR technique was employed to evaluate the affinity of remaining peptides towards the MBP, and the results are collected in Table 2. Dose response assays clearly revealed the recognition of 9 out of 11 designed peptides towards the protein. Exceptions are MBP-10 and MBP-11 that did not give valuable signal variations in the explored concentration range (Figure CD and Figure CE in **S1 File**). MBP-8 provided a dissociation constant in the micromolar range: K_D_ = 200±5 μM, estimated by applying a 1:1 Langmuir model for the interaction in the evaluation of kinetic parameters. MBP-1, MBP-3 and MBP-6 gave saturated signals and for them the fitting of RU_max_ values vs peptides concentrations provided K_D_ values in the millimolar range (4.2±1.4, 1.5±0.2, 1.3±0.5 mM, Figure DA-C in **S1 File**). The other peptides showed dose-response signal variations without reaching saturation (Figure C in **S1 File**), due to their tendency to aggregate. Further, MBP-8 at 200μM was also co-injected with maltose in a 1:100 dramatically decrease of the RU signal upon the presence of maltose, as formerly observed for MBP-9, while MBP-1 and MBP-6 were tested for their specificity by MS-ESI with Ovalbumin and Neuroserpin: also in these cases quite small amounts of the complexes were detected at 10-fold molar excess of the ligand confirming a strong specificity in the sequence able to recognize MBP (data not shown).

**Fig 7:**
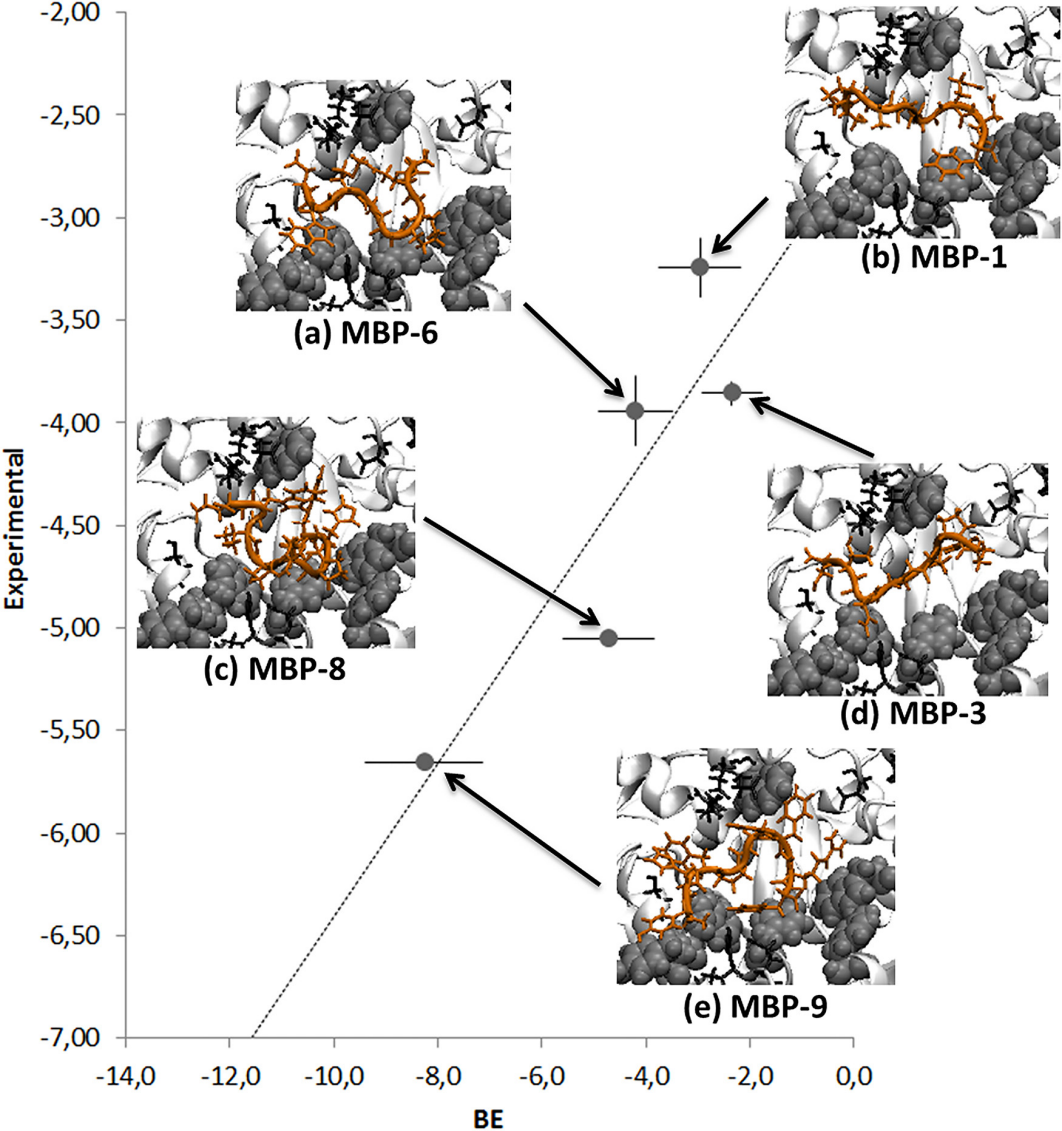
Comparison between the experimental binding and the MC+Vina calculated BE. The BE errorbars are standard errors of the means over 10 samples, while the experimental errobars are calculated as σ_ΔG_ = 0.434(σ_kD_/k_D_),. Docking into maltose site of peptides: (a) MBP-6, (b) MBP-1, (c) MBP-8, (d) MBP-3, (e) MBP-9. Highlighted with their Van der Waals spheres the MBP aromatic side chains involved in the binding (Tyr 155 219 34, Phe 337, Trp 62 340 230) and in black those capable of hydrogen bonding (Ser 337, Asp 14, Arg 66 344, Lys 297, Glu 153 44 45).

By converting the binding affinities in units of energy, using ΔG = RTln(k_D_) with RT = 0.593kcal/mol with error defined as σ_ΔG_ = 0.434(σ_kD_/k_D_) where σ_kD_ is the experimental, it is possible to compare the measured binding affinities with the BE calculated in Sec.2.2 (Fig 7). In the limit of a very small dataset, the comparison shows a good correlation.

The inspection of the protein maltose binding site revealed the presence of several aromatic and hydrogen bonding residues besides of Met 330 (see the insets of Fig 7). The formers allow for multiple π-π interactions between the protein and the peptides aromatic rings. This is particularly evident for MBP-9 which is endowed with a pronounced aromatic character. But the aromaticity itself is not sufficient to guarantee binding, since it is pronounced for binding as well as for “non-binding” sequences. Indeed the comparison between the amino acidic composition of the most potent binders (MBP-8, -9) and the non-binding sequences (MBP-10, -11), showed that even if they share common chemical features only the binding peptides bear Proline residues. This iminoacid, in the middle of the sequences, seems to ensure a conformational turn that could better accommodate peptides in the maltose site, probably aiding π-stacking interactions among protein and aromatic side-chains of peptide sequences.

## Discussion and Conclusions

We have computationally generated a set of octa-peptides targeting the maltose site of MBP with an algorithm previously employed successfully in the framework of drug recognition. Peptides were screened with a docking based algorithm, allowing the selection of the best candidate binder out of a number of possibilities. SPR, ESI-MS and fluorescence based assays confirmed its 1:1 stochiometry, its selectivity towards the binding site they have been designed for and leading to a binding affinity in the micromolar range.

However, current docking algorithms while pinpointing possible binders and predicting their bound conformation turn out to be unable to rank and estimate the binding energy between the peptides and the MBP and to discern between binders and non-binders. For instance, the original Vina binding energy is reasonably accurate for small molecules: the maltotriose with MBP open configuration scores -7.1kcal/mol and -8.0kcal/mol for the closed configuration to be compared to the experimental values of -8.4kcal/mol[45] and -9.3kcal/mol[46]. Vina+MC is accurate for very short peptides, for instance KAK+OPPA with the MC+Vina code scores -9.5±0.6 kcal/mol (the experimentally determined value is -9.8kcal/mol [47]). It is also possible to reproduce the measured binding affinity of -9.7kcal/mol obtained for the octapeptide of Ref.[37] with calculated average value of 7.1±1.1kcal/mol (with a minimum at -10.2 kcal/mol). However Vina, as well as the others Vina-based protocols, is orders of magnitude far from the experimentally evaluated binding energies for longer peptides.

While this is an important obstacle for the progress of the *in silico* optimization of peptide-based binders, we have delineated a new route. In order to match the experimental binding affinities and computational values, we have demonstrated that it is crucial to consider a negative control in the estimation of the entropic contribution of the global binding energy. Indeed we have redefined this contribution as the “binding” energy of the peptide towards an experimentally determined non-binding configuration. ESI-MS and SPR experiments, showed that the peptides do not form a 1:1 complex with MBP when an excess of maltose is present, indicating that they are specific for MBP in its maltose-free conformation and that they are unable to bind to the MBP maltose-containing-closed conformation, making the maltose-containing-closed conformation a reliable negative control. Further, since the structure of the two MBP globular domains is unaffected by the presence of maltose, we chose the multistranded region connecting them as control for peptide aspecific binding even if we did not exclude *a priori* that aspecific interactions could occur in the globular lobes.

While in the proposed work we assess the limitations of the docking based automated evolution of protein binding peptides, the redefinition of the ligand binding energy provides an intriguing direction for the computational evolution and screening of peptides with reliable predicted binding affinities for proteins.

## Methods

### Computational

For molecular modelling and docking we used the ProteinDataBank structures 1OMP[48] and 3MBP[46]. The docking protocol, implemented in a bash script, is based on a combination of Modeler 9.11[49], AutoDock Tools 1.5.4, and AutoDock Vina 1.1.0 [18]. All dockings were performed in a box of size 25x25x25Å, to constrain the peptides in the MBP binding pocket, with exhaustiveness 10, and energy_range 4. The other parameters have been set to their default values. For MBP the box was centered on the sulfur atom of Met330. The starting configuration for all the Vina-based docking runs was a linear poly-alanine. AutoDock Vina was run 10 times obtaining 90 configurations, MC+Vina 10 times for 100 steps obtaining 100 end-simulation configurations. All the figures throughout the text were generated with VMD.

### Protein Preparation and characterization of His MBP

This protein was expressed, purified, and characterized as reported in Figure B in **S1 File** [50]

### Peptide Synthesis

Reagents (Fmoc-protected amino acids and resins, activation and deprotection reagents) from Novabiochem (Laufelfingen, Switzerland) and InBios (Napoli, Italy); Solvents and HPLC analyses from Romil (Dublin, Ireland); reversed phase columns for peptide analysis and LC-MS system from ThermoFisher (Milan, Italy). Solid phase peptide syntheses were performed on a fully automated multichannel peptide synthesizer Syro I (Multisynthech, Germany). Preparative RP-HPLC was carried out on a Shimadzu LC-8A, equipped with a SPD-M10 AV detector and with a Phenomenex C18 Jupiter column (50x22 mm ID; 10 μm). LC-MS analyses were carried out on a LCQ DECA XP Ion Trap mass spectrometer equipped with a OPTON ESI source, operating at 4.2 kV needle voltage and 320°C with a complete Surveyor HPLC system, comprised of MS pump, an autosampler and a photo diode array (PDA), and Narrow bore 50x2 mm C18 BioBasic LC-MS columns. Peptides were synthesized employing the solid phase method on a 50 μmol scale following standard Fmoc strategies[51] with Rink-amide resin (substitution 0.5 mmol/g) solid support. The amino acids were activated with HBTU/Oxyme /DIEA (1:1:2), and the Fmoc deprotection was achieved by using a 20% (v/v) piperidine solution in DMF. All couplings were performed for 15 min and deprotections for 10 min. Finally the peptides were removed from the resin by treatment with a TFA:TIS:H2O (90:5:5, v/v/v) mixture for 90 min at room temperature. Crude peptides were then precipitated in cold ether, dissolved in a water/acetonitrile (1:1, v/v) and lyophilized. Products were purified by RP-HPLC applying a linear gradient of 0.1% TFA CH_3_CN in 0.1% TFA water from 1% to 30% or 5% to 70% depending on the hydrophobicity over 13 min using a semi-preparative 2.2x5 cm C18 column at a flow rate of 15 mL/min. Peptides purity and identity were confirmed by LC-MS. Purified peptides were lyophilized and stored at -20°C until use.

### ESI-MS analysis of protein-peptides complexes

ESI-MS analysis were carried out on a hybrid quadrupole time-of-flight mass spectrometer (QSTAR Elite, AB-Sciex, ForsterCity, CA, USA), which is equipped with nano-ESI source [52]. The samples were infused by metal-coated borosilicate capillaries, with emitter tips of 1μm internal diameter (Proxeon, Odense, Denmark), and the instrumental setting was: declustering potential (DP) +60 V, ion spray voltage +1.1/+1.2 kV and curtain-gas pressure 20psi. Both the sample source and the instrument interface were kept at room temperature. The spectra were recorded under nondenaturing conditions (50 mM ammonium acetate pH 7). The protein complexes with peptides were prepared by mixing 20μM protein solution with an equimolar amount of ligand.

### Surface Plasmon Resonance

The interactions between the protein and computationally optimized peptides were measured using the BIAcore 3000 (GE Healthcare Milano, Italy). MBP was immobilized at a concentration of 100 μg/mL in 10 mM acetate buffer pH 5 (flow rate 5 μL/min, time injection 7 min) on a CM5 Biacore sensor chip, using EDC/NHS chemistry following the manufacturer’s instructions. Residual reactive groups were deactivated by treatment with 1 M ethanolamine hydrochloride, pH 8.5. The reference channel was activated with EDC/NHS and deactivated with ethanolamine. The binding assays were carried out at 20 μL /min at 25°C, with 4.5 min contact-time. Peptides were diluted in the HBS running buffer (10 mM Hepes, 150 mM NaCl, 3 mM EDTA, pH 7.4). Analyte injections were performed at the indicated concentrations. The sensor surface was regenerated by using 1–3 washes of 10mM NaOH for 1 minute. The association phase (kon) was followed for 250s, whereas the dissociation phase (koff) was followed for 300 s. The instrument BIAevaluation analysis package (version 4.1, GE Healthcare, Milano, Italy) was used to subtract the signal of the reference channel. Conversely, an affinity steady state model was applied to fit the RU_max_ data versus peptides concentrations and fitting was performed with GraphPad Prism v4.00 using the one-site binding equation [53,54].

### Fluorescence binding analysis

The data were acquired at 25.0°C, using an excitation wavelength of 298.0 nm and a fluorescence emission wavelength ranging from 300 to 400 nm. The acquisition parameters were set as follows: excitation and emission slits at 5 nm; 120 nm/min scan rate; 1.00 nm data interval averaging time at 0.500 s, PMT voltage at “high”. The fluorescence values recorded at 333 nm were extracted, and transformed to -Δfluorescence which was obtained by subtracting them to the emission fluorescence intensity of the ligand-free protein, and, then, plotted against the peptide concentration [55]. MBP was used at the concentration of 14 μM and incubated in the presence of increasing concentrations of MBP-8 ranging from 0 to 350 μM. Experiments were carried out in duplicates. A control assay was carried out employing as titrant the buffer to assess that the dilution effect was under 3%, not affecting the results.

## Supporting Information

S1 File Docking, Protein Preparation and characterization of His-tagged MBP (Expression and Purification, CD Measurements, ESI-MS), Supporting binding data. (DOCX)

## References

[1] Ball HJ, Finlay D (1998) Diagnostic application of monoclonal antibody (MAb)-based sandwich ELISAs. Methods Mol Biol 104: 127–132. 9711648 10.1385/0-89603-525-5:127

[2] Carter PB, Beegle KH, Gebhard DH (1986) Monoclonal antibodies. Clinical uses and potential. Vet Clin North Am Small Anim Pract 16: 1171–1179. 3024378 10.1016/s0195-5616(86)50135-2

[3] Goldberg ME, Djavadi-Ohaniance L (1993) Methods for measurement of antibody/antigen affinity based on ELISA and RIA. Curr Opin Immunol 5: 278–281. 8507406 10.1016/0952-7915(93)90018-n

[4] Harlow E, Lane D (1988) Antibodies: A Laboratory Manual: Cold Spring Harbor Laboratory, New York.

[5] Zhou P, Wang C, Ren Y, Yang C, Tian F (2013) Computational Peptidology: A New and Promising Approach to Therapeutic Peptide Design. Current Medicinal Chemistry 20: 1985–1996. 23317161 10.2174/0929867311320150005

[6] Zeng J, Nheu T, Zorzet A, Catimel B, Nice E, Maruta H, et al (2001) Design of inhibitors of Ras-Raf interaction using a computational combinatorial algorithm. Protein Engineering 14: 39–45. 11287677 10.1093/protein/14.1.39

[7] Zhang Z, Zhu W, Kodadek T (2000) Selection and application of peptide-binding peptides. Nat Biotech 18: 71–74. 10.1038/71951 10625395

[8] Schneider S, Buchert M, Georgiev O, Catimel B, Halford M, Stacker SA, et al (1999) Mutagenesis and selection of PDZ domains that bind new protein targets. Nat Biotech 17: 170–175. 10.1038/6172 10052354

[9] Anisimov VM, Ziemys A, Kizhake S, Yuan ZY, Natarajan A, Cavasotto CN (2011) Computational and experimental studies of the interaction between phospho-peptides and the C-terminal domain of BRCA1. Journal of Computer-Aided Molecular Design 25: 1071–1084. 10.1007/s10822-011-9484-3 22086652 PMC3400539

[10] Smith GP, Petrenko VA (1997) Phage display. Chemical Reviews 97: 391–410. 11848876 10.1021/cr960065d

[11] Scott JK, Smith GP (1990) Searching for peptide ligands with an epitope library. Science 249: 386–390. 1696028 10.1126/science.1696028

[12] Li Y, Moysey R, Molloy PE, Vuidepot AL, Mahon T, Baston E, et al (2005) Directed evolution of human T-cell receptors with picomolar affinities by phage display. Nature Biotechnology 23: 349–354. 15723046 10.1038/nbt1070

[13] London N, Raveh B, Schueler-Furman O (2013) Peptide docking and structure-based characterization of peptide binding: from knowledge to know-how. Current Opinion in Structural Biology 23: 894–902. 10.1016/j.sbi.2013.07.006 24138780

[14] London N, Raveh B, Cohen E, Fathi G, Schueler-Furman O (2011) Rosetta FlexPepDock web server-high resolution modeling of peptide-protein interactions. Nucleic Acids Research 39: W249–W253. 10.1093/nar/gkr431 21622962 PMC3125795

[15] Raveh B, London N, Schueler-Furman O (2010) Sub-angstrom modeling of complexes between flexible peptides and globular proteins. Proteins-Structure Function and Bioinformatics 78: 2029–2040. 10.1002/prot.22716 20455260

[16] Donsky E, Wolfson HJ (2011) PepCrawler: a fast RRT-based algorithm for high-resolution refinement and binding affinity estimation of peptide inhibitors. Bioinformatics 27: 2836–2842. 10.1093/bioinformatics/btr498 21880702

[17] Dominguez C, Boelens R, Bonvin AMJJ (2003) HADDOCK: A Protein−Protein Docking Approach Based on Biochemical or Biophysical Information. Journal of the American Chemical Society 125: 1731–1737. 12580598 10.1021/ja026939x

[18] Trott O, Olson AJ (2010) Software News and Update AutoDock Vina: Improving the Speed and Accuracy of Docking with a New Scoring Function, Efficient Optimization, and Multithreading. Journal of Computational Chemistry 31: 455–461. 10.1002/jcc.21334 19499576 PMC3041641

[19] Li HL, Li CL, Gui CS, Luo XM, Chen KX, Shen JH, et al (2004) GAsDock: a new approach for rapid flexible docking based on an improved multi-population genetic algorithm. Bioorganic & Medicinal Chemistry Letters 14: 4671–4676. 15324886 10.1016/j.bmcl.2004.06.091

[20] Zheng F, Jewell H, Fitzpatrick J, Zhang J, Mierke DF, Grigoryan G (2015) Computational Design of Selective Peptides to Discriminate between Similar PDZ Domains in an Oncogenic Pathway. Journal of Molecular Biology 427: 491–510. 10.1016/j.jmb.2014.10.014 25451599 PMC4970318

[21] London N, Lamphear CL, Hougland JL, Fierke CA, Schueler-Furman O (2011) Identification of a Novel Class of Farnesylation Targets by Structure-Based Modeling of Binding Specificity. PLoS computational biology 7. 10.1371/journal.pcbi.1002170 PMC3188499 21998565

[22] Smith CA, Kortemme T (2010) Structure-Based Prediction of the Peptide Sequence Space Recognized by Natural and Synthetic PDZ Domains. Journal of Molecular Biology 402: 460–474. 10.1016/j.jmb.2010.07.032 20654621

[23] Deaven DM, Tit N, Morris JR, Ho KM (1996) Structural optimization of Lennard-Jones clusters by a genetic algorithm. Chemical Physics Letters 256: 195–200.

[24] Deaven DM, Ho KM (1995) Molecular Geometry Optimization with a Genetic Algorithm. Physical Review Letters 75: 288–291. 10059656 10.1103/PhysRevLett.75.288

[25] Moret MA, Pascutti PG, Bisch PM, Mundim KC (1998) Stochastic molecular optimization using generalized simulated annealing. Journal of Computational Chemistry 19: 647–657.

[26] Kirkpatrick S, Gelatt CD Jr., Vecchi MP (1983) Optimization by simulated annealing. Science (New York, NY) 220: 671–680. 10.1126/science.220.4598.671 17813860

[27] Hohl D, Jones RO, Car R, Parrinello M (1988) Structure of sulfur clusters using simulated annealing: S2 to S1 3. Journal of Chemical Physics 89: 6823–6835.

[28] Sugita Y, Okamoto Y (1999) Replica-exchange molecular dynamics method for protein folding. Chemical Physics Letters 314: 141–151.

[29] Fortuna S, Troisi A (2010) Agent-Based Modeling for the 2D Molecular Self-Organization of Realistic Molecules. The Journal of Physical Chemistry B 114: 10151–10159. 10.1021/jp103950m 20684638

[30] Fortuna S, Troisi A (2009) An Artificial Intelligence Approach for Modeling Molecular Self-assembly: Agent-based Simulations of Rigid Molecules. The Journal of Physical Chemistry B 113: 9877–9885. 10.1021/jp9030442 19569637

[31] Huang S-Y, Grinter SZ, Zou X (2010) Scoring functions and their evaluation methods for protein-ligand docking: recent advances and future directions. Physical Chemistry Chemical Physics 12: 12899–12908. 10.1039/c0cp00151a 20730182 PMC11103779

[32] Yagi Y, Terada K, Noma T, Ikebukuro K, Sode K (2007) In silico panning for a non-competitive peptide inhibitor. Bmc Bioinformatics 8. 10.1186/1471-2105-8-11 PMC1781467 17222344

[33] Abe K, Kobayashi N, Sode K, Ikebukuro K (2007) Peptide ligand screening of alpha-synuclein aggregation modulators by in silico panning. Bmc Bioinformatics 8. 10.1186/1471-2105-8-451 PMC2244645 18005454

[34] Belda I, Madurga S, Llora X, Martinell M, Tarrago T, Piqueras MG, et al (2005) ENPDA: an evolutionary structure-based de novo peptide design algorithm. Journal of Computer-Aided Molecular Design 19: 585–601. 16267689 10.1007/s10822-005-9015-1

[35] Heurich M, Altintas Z, Tothill IE (2013) Computational Design of Peptide Ligands for Ochratoxin A. Toxins 5: 1202–1218. 10.3390/toxins5061202 23793075 PMC3717777

[36] Earl DJ, Deem MW (2005) Parallel tempering: Theory, applications, and new perspectives. Physical Chemistry Chemical Physics 7: 3910–3916. 19810318 10.1039/b509983h

[37] Hong-Enriquez RP, Pavan S, Benedetti F, Tossi A, Savoini A, Berti F, et al (2012) Designing Short Peptides with High Affinity for Organic Molecules: A Combined Docking, Molecular Dynamics, And Monte Carlo Approach. Journal of Chemical Theory and Computation 8: 1121–1128. 26593371 10.1021/ct200873y

[38] Medintz IL, Goldman ER, Lassman ME, Mauro JM (2003) A Fluorescence Resonance Energy Transfer Sensor Based on Maltose Binding Protein. Bioconjugate Chemistry 14: 909–918. 13129393 10.1021/bc020062+

[39] Quiocho FA, Ledvina PS (1996) Atomic structure and specificity of bacterial periplasmic receptors for active transport and chemotaxis: Variation of common themes. Molecular Microbiology 20: 17–25. 8861200 10.1111/j.1365-2958.1996.tb02484.x

[40] Bucher D, Grant BJ, Markwick PR, McCammon JA (2011) Accessing a hidden conformation of the maltose binding protein using accelerated molecular dynamics. PLoS computational biology 7: e1002034 10.1371/journal.pcbi.1002034 21533070 PMC3080849

[41] Fukami-Kobayashi K, Tateno Y, Nishikawa K (1999) Domain dislocation: a change of core structure in periplasmic binding proteins in their evolutionary history. Journal of Molecular Biology 286: 279–290. 9931266 10.1006/jmbi.1998.2454

[42] Stockner T, Vogel HJ, Tieleman DP (2005) A salt-bridge motif involved in ligand binding and large-scale domain motions of the maltose-binding protein. Biophysical Journal 89: 3362–3371. 16143635 10.1529/biophysj.105.069443 PMC1366833

[43] Gilardi G, Mei G, Rosato N, Agrò AF, Cass AE (1997) Spectroscopic properties of an engineered maltose binding protein. Protein Engineering 10: 479–486. 9215565 10.1093/protein/10.5.479

[44] Miller DM 3rd, Olson JS, Pflugrath JW, Quiocho FA (1983) Rates of ligand binding to periplasmic proteins involved in bacterial transport and chemotaxis. The Journal of biological chemistry 258: 13665–13672. 6358208

[45] Telmer PG, Shilton BH (2003) Insights into the conformational equilibria of maltose-binding protein by analysis of high affinity mutants. Journal of Biological Chemistry 278: 34555–34567. 12794084 10.1074/jbc.M301004200

[46] Quiocho FA, Spurlino JC, Rodseth LE (1997) Extensive features of tight oligosaccharide binding revealed in high-resolution structures of the maltodextrin transport/chemosensory receptor. Structure 5: 997–1015. 9309217 10.1016/s0969-2126(97)00253-0

[47] Sleigh SH, Seavers PR, Wilkinson AJ, Ladbury JE, Tame JRH (1999) Crystallographic and Calorimetric Analysis of Peptide Binding to OppA Protein. Journal of Molecular Biology 291: 393–415. 10438628 10.1006/jmbi.1999.2929

[48] Sharff AJ, Rodseth LE, Spurlino JC, Quiocho FA (1992) Crystallographic evidence of a large ligand-induced hinge-twist motion between the two domains of the maltodextrin binding protein involved in active transport and chemotaxis. Biochemistry 31: 10657–10663. 1420181 10.1021/bi00159a003

[49] Šali A, Blundell TL (1993) Comparative Protein Modelling by Satisfaction of Spatial Restraints. Journal of Molecular Biology 234: 779–815. 8254673 10.1006/jmbi.1993.1626

[50] Balan A, de Souza CS, Moutran A, Ferreira RC, Franco CS, Ramos CH, et al Purification and in vitro characterization of the maltose-binding protein of the plant pathogen Xanthomonas citri. Protein Expr Purif: 2005 10;2043(2002):2103–2010. 10.1016/j.pep.2005.03.018 16139753

[51] Fields GB, Noble RL (1990) Solid phase peptide synthesis utilizing 9-fluorenylmethoxycarbonyl amino acids. International Journal of Peptide and Protein Research 35: 161–214. 2191922 10.1111/j.1399-3011.1990.tb00939.x

[52] Grandori R, Santambrogio C, Brocca S, Invernizzi G, Lotti M (2009) Electrospray-ionization mass spectrometry as a tool for fast screening of protein structural properties. Biotechnology Journal 4: 73–87. 10.1002/biot.200800250 19156745

[53] Scognamiglio PL, Di Natale C, Leone M, Poletto M, Vitagliano L, Tell G, et al (2014) G-quadruplex DNA recognition by nucleophosmin: New insights from protein dissection. Biochimica et biophysica acta 1840: 2050–2059. 10.1016/j.bbagen.2014.02.017 24576674

[54] Poletto M, Vascotto C, Scognamiglio PL, Lirussi L, Marasco D, Tell G (2013) Role of the unstructured N-terminal domain of the hAPE1 (human apurinic/apyrimidinic endonuclease 1) in the modulation of its interaction with nucleic acids and NPM1 (nucleophosmin). Biochemical Journal 452: 545–557. 10.1042/BJ20121277 23544830

[55] Williamson MP (2013) Using chemical shift perturbation to characterise ligand binding. Progress in Nuclear Magnetic Resonance Spectroscopy 73: 1–16. 10.1016/j.pnmrs.2013.02.001 23962882

