# Correction: *In Silico* Generation of Peptides by Replica Exchange Monte Carlo: Docking-Based Optimization of Maltose-Binding-Protein Ligands

**DOI:** 10.1371/journal.pone.0165349

**Published:** 2016-10-18

**Authors:** Anna Russo, Pasqualina Liana Scognamiglio, Rolando Pablo Hong Enriquez, Carlo Santambrogio, Rita Grandori, Daniela Marasco, Antonio Giordano, Giacinto Scoles, Sara Fortuna

The following information is missing from the funding section: This study was partially supported by the European Fund for Regional Development—Cross-Border Cooperation Programme Italy-Slovenia 2007–2013, (Project PROTEO, Code N. CB166).
