# Supplementary material for: In Silico Generation of Peptides by Replica Exchange Monte Carlo: Docking-Based Optimization of Maltose-Binding-Protein Ligands
Source: PLoS One. 2015 Aug 7;10(8):e0133571. doi: 10.1371/journal.pone.0133571 (PMC4529233; doi:10.1371/journal.pone.0133571)
Supplement: S1 File — (DOCX) [file pone.0133571.s001.docx]

***In silico* generation of peptides by replica exchange Monte Carlo: Docking-based optimization of maltose-binding-protein ligands**

##### *Anna Russo^1,2,¶^ , Pasqualina Liana Scognamiglio^3,¶^ , Rolando P. Hong Enriquez^4^, Carlo Santambrogio^5^, Rita Grandori^5^, Daniela Marasco^3^*^,+^*, Antonio Giordano^6,7^, Giacinto Scoles^1,8^ and Sara Fortuna^1,^**

##### *^1^Department of Medical and Biological Sciences, University of Udine, Piazzale Kolbe, 4 - 33100 Udine – Italy*

##### *^2^Department of Medical Biotechnology, University of Siena, Policlinico Le Scotte, Viale Bracci, 2 – 53100 Siena - Italy*

##### *^3^Department of Pharmacy, CIRPEB: Centro Interuniversitario di Ricerca sui Peptidi Bioattivi- University of Naples “Federico II”, DFM-Scarl, 80134, Naples, Italy.*

##### *^4^Department of Drug Discovery and Development, Italian Institute of Technology (IIT), Genova, Italy*

##### *^5^Department of Biotechnology and Biosciences, University of Milano-Bicocca, Piazza della Scienza, 2 - 20126 Milan – Italy*

##### *^6^Sbarro Institute for Cancer Research and Molecular Medicine & Center for Biotechnology Temple University Philadelphia, PA USA*

##### *^7^Department of Medicine, Surgery & Neuroscience University of Siena, Strada delle Scotte n. 6, 53100 Siena*

##### *^8^Department of Biology, Temple University, Philadelphia (PA), USA*

##### *^¶^These authors equally contributed to this work*

Corresponding author: E-mail: sara.fortuna@uniud.it

+ E-mail: daniela.marasco@unina.it

**Docking**

Autodock Vina was run for 10 times obtaining 90 configurations, MC+Vina for 10 times for 100 steps obtaining 100 end-simulation configurations, and REMC+Vina for 3 times for 100 steps obtaining 90 low-energy configurations. The lowest energy configuration obtained with MC+Vina was the starting configuration for FlexPepDock run in only one instance leading to 10 configurations which were then rescored with Autodock Vina (Table S1). The Rosetta FlexPepDock web server [[1](#_ENREF_1), [2](#_ENREF_2)]] was used to generate 100 low resolution structures and 10 high resolution structures. The lowest energy configurations are shown if Figure S1.


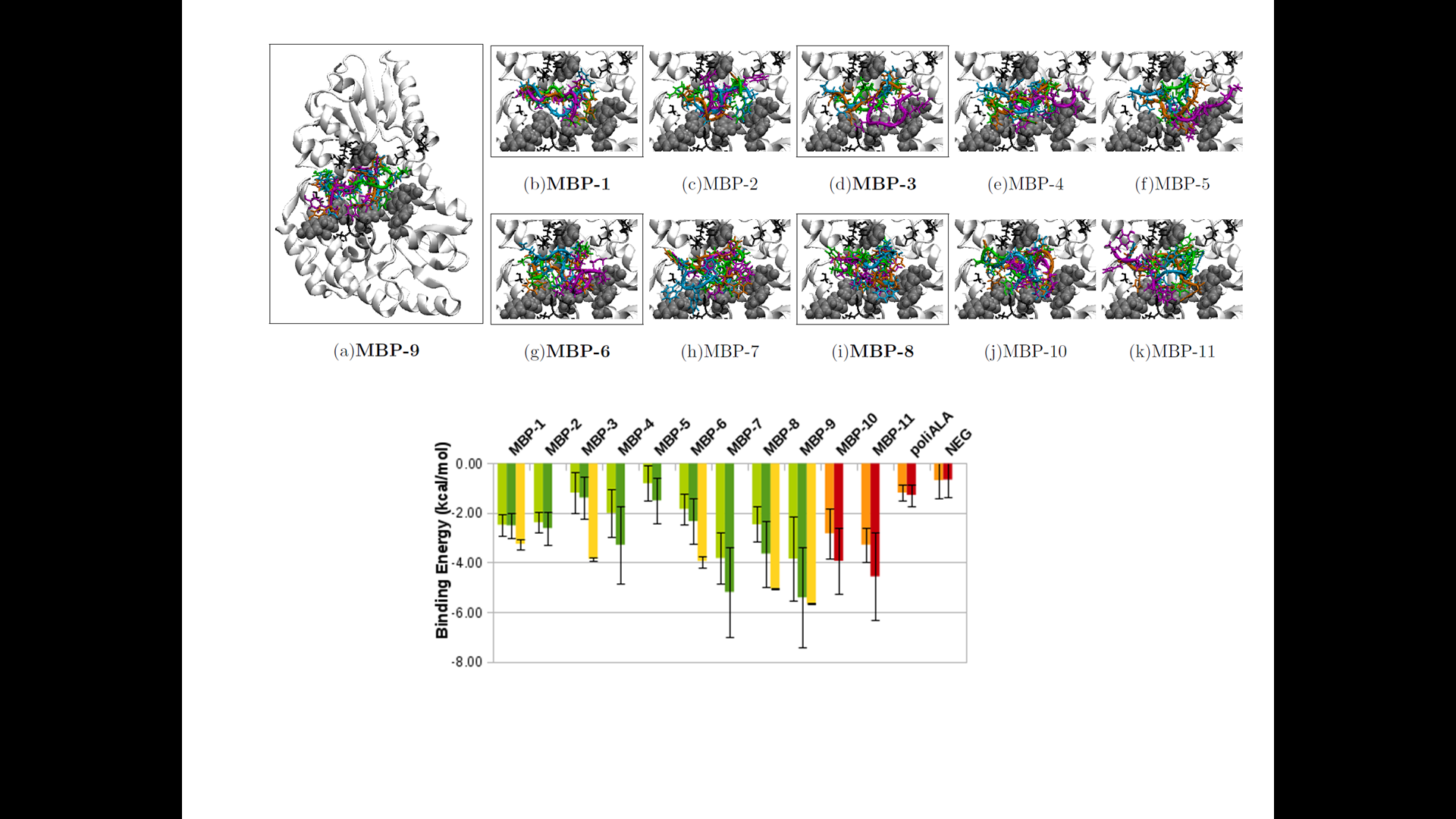


**Figure A.** Lowest energy end-simulation configurations for each computationally generated peptide by using different docking protocols: Vina (green), MC+Vina (orange), REMC+Vina (blue), FlexPepDock (pink). Highlighted with their Van der Waals spheres the MBP aromatic side chains involved in the binding (TYR 155 219 34, PHE 337, TRP 62 340 230) and in black those capable of hydrogen bonding (SER 337, ASP 14, ARG 66 344, LYS 297, GLU 153 44 45).

**Table A.** Lowest energy configuration docking scores calculated with Autodock Vina and in parenthesis the same scores averaged over multiple configurations, highlighted in italics the non-binders. All the values are in kcal/mol.

| peptide | sequence | Vina | MC+Vina | REMC+Vina | FlexPepDock |
| --- | --- | --- | --- | --- | --- |
| MBP-1 | SPAGGQDF | -8.6 (-7.8±0.6) | -17.7 (-14.7±1.6) | -14.9 | -14.0 (-12.8±1.0) |
| MBP-2 | WGTNGGTR | -8.7 (-7.7±0.5) | -17.0 (-14.1±1.4) | -15.6 | -14.5 (-13.6±0.7) |
| MBP-3 | APRGGNTS | -7.8 (-7.0±0.6) | -15.6 (-13.8±1.2) | -14.9 | -14.7 (-12.0±1.7) |
| MBP-4 | PQYPPHDN | -9.2 (-8.0±0.6) | -17.0 (-15.1±1.0) | -17.3 | -15.8 (-13.4±1.6) |
| MBP-5 | GLPKPGGN | -7.3 (-6.6±0.3) | -13.4 (-12.5±0.5) | -13.3 | -12.1 (-10.4±1.1) |
| MBP-6 | PQKGGMWD | -7.6 (-7.1±0.4) | -16.9 (-14.9±1.3) | -16.1 | -16.7 (-12.1±2.6) |
| MBP-7 | WSPNFWWR | -9.3 (-8.3±0.6) | -20.2 (-16.8±1.5) | -18.0 | -22.3 (-19.7±1.8) |
| MBP-8 | WHPRPVWE | -9.0 (-8.3±0.3) | -16.0 (-14.8±1.1) | -18.1 | -16.9 (-14.6±1.8) |
| MBP-9 | YHFPYFRF | -10.2 (-8.9±0.7) | -20.1 (-18.7±1.1) | -19.3 | -21.5 (-19.3±2.0) |
| *MBP-10* | *YGDGYFRF* | *-9.0 (-8.1±0.4)* | *-18.8 (-15.1±1.4)* | *-17.1* | *-19.6 (-16.4±2.7)* |
| *MBP-11* | *YHDGYFRF* | *-8.8 (-8.1±0.5)* | *-20.0 (-16.2±2.4)* | *-19.2* | *-18.7 (-15.8±1.8)* |
| *poliALA* | *AAAAAAAA* | *-7.8 (-7.1±0.3)* | *-12.3 (-11.0±1.0)* | *-11.9* | *-12.2 (-10.8±1.3)* |
| *NEG* | *AAARRAAA* | *-7.5 (-6.9±0.4)* | *-15.3 (-13.3±1.1)* | *-14.3* | *-15.6 (-12.3±1.7)* |

**Protein Preparation and characterization of His-tagged MBP**

**Expression and Purification**

The plasmid pETM-44 encoding for N-terminus his-tagged MBP were from EMBL. E. coli BL21(DE3) strain was purchased from Agilent Technologies. Recombinant histidine-tagged Maltose Binding Protein, was produced by transforming pETM44 vector into E. coli BL21(DE3) strain according to the manufacturer's protocol. Cultures were grown aerobically with shaking (220rpm) at 37°C in Erlenmeyer flasks containing LB medium with 50µg/ml kanamycin, until OD_600_ reached 0.6 value. Then the cultures were induced with IPTG 0.1mM aerobically for 4 h with shaking (220rpm) at 37°C (figure S2-A).[[3](#_ENREF_3)]

Cells were harvested by centrifugation at 5000rpm for 15 minutes at 4°C (Sorvall RC-B5, rotor G53) and stored at –80°C. Cell pellets were resuspended in buffer 1 (20mM sodium phosphate, pH 7.4, 500mM NaCl) containing 20mM imidazole supplemented with a protease inhibitor cocktail (complete EDTA-free Roche). The suspension was incubated on ice for 1h with 1 mg∕ml lysozyme (Sigma), sonicated, and centrifuged at 14000 rpm for 30 min at 4°C (Sorvall Evolution RC, rotor SS-34). The protein was purified by affinity chromatography on a HisTrap FF column by using AKTA Purifier UPC10 (GeHealthCare). The unclarified cell lysate supernatant was applied to the column, previously equilibrated with binding buffer (buffer1+20mM imidazole). The column was rinsed with binding buffer and unbound fractions were collected, then the one-step elution of HisMBP was performed using buffer 1+500mM imidazole (figure S2-B).

Fractions were analyzed by 12% SDS/PAGE stained by silver development, pooled (figure S2-C), and dialyzed against 50mM sodium phosphate, pH 7.4 at 4°C, by using 10kDa cut-off snake skin dialysis membrane (Pierce). The Bradford protein assay was used to measure protein concentration.

**CD Measurements**

CD measurements were performed on a J-815 spectropolarimeter (Jasco Corp., Tokyo, Japan). Far-UV (190 to 260 nm) CD spectra were recorded for 1 μM protein in a 2.0mm pathlength quarzt cell (Hellma) at a scan speed of 20nm min^-1^, with a resolution of 0.1nm. Each spectrum was an average of two consecutive scans, and buffer scans were accumulated and subtracted from protein spectra (figure S2-D). The CD spectrum of MBP is characteristic of an α-helical with minima at 208 and 222.[[4](#_ENREF_4)]

**ESI-MS**

The MBP activity was evaluated by ESI-MS. The protein spectrum was recorded under nondenaturing conditions (50 mM ammonium acetate pH 7) at the concentration of 3µM. The protein complexes with maltose were prepared by mixing 10-fold molar excess of ligand and allowing reaching the binding equilibrium in 5 minutes at room temperature. The protein gives rise to a narrow charge-state distribution (only 4 peaks) centered on the 14+ charge state. This is the typical behavior of compact, folded structures. Similar charge-state distributions are detected in the presence of maltose (figure S2-E), where new peaks corresponding to MBP-maltose complexes appear close to the previous ones.

**Figure B. Characterization of His tagged MBP.** (A) SDS-PAGE of lysate of expressed HisMBP in E. coli BL21(DE3) . Lane 1: unstained markers, lane 2: whole-cell extracts of non-induced cultures, lane 3: whole-cell extracts of induced cultures. (B) FPLC chromatogram of His-tagged MBP purification on a HisTrap FF column. (C) Analysis of protein purification: Lane 1: unstained markers, lane 2: unbound, lane 3: eluted protein. (D) CD spectrum of MBP 1 μM in sodium phosphate 50mM pH 7.2. (E) ESI-MS spectrum of 3μM MBP and 3μM Maltose in 50 mM ammonium acetate pH7, DP 60V. The peaks corresponding to apoMBP (○) and MBP:maltose 1:1 complex (●) are labeled.**Supporting binding data**

**Figure C. Binding of MBP-2, 4, 7, 10, 11 peptide toward MBP protein through SPR.** Overlay of sensorgrams for the interaction between immobilized MBP on a CM5 chip with an immobilization level of 4900 RU and (A) MBP2, (B) MBP4, (C) MBP7, (D) MBP10, (E) MBP11. Dose response behavior were exhibited by those peptides, except MBP-10 and MBP-11 that did not give valuable signal variations in the explored concentration range.

**Figure D. Binding of MBP1, MBP3 and MBP6 peptides toward MBP protein through SPR assay**.

Indicated peptides gave saturated signals in a dose response assays. The RU_max_ values related to increasing concentrations of peptide were fitted according to a single exponential binding model with 1:1 stoichiometry (A) for MBP1 (0-3 mM), (B) for MBP3 (0-9mM) and (C) for MBP6 (0-4 mM).

**References**

1. London, N., et al., *Rosetta FlexPepDock web server-high resolution modeling of peptide-protein interactions.* Nucleic Acids Research, 2011. **39**: p. W249-W253.

2. Raveh, B., N. London, and O. Schueler-Furman, *Sub-angstrom modeling of complexes between flexible peptides and globular proteins.* Proteins-Structure Function and Bioinformatics, 2010. **78**(9): p. 2029-2040.

3. Balan, A., et al., *Purification and in vitro characterization of the maltose-binding protein of the plant pathogen Xanthomonas citri.* Protein Expression and Purification, 2005. **43**(2): p. 103-110.

4. Quiocho, F.A., J.C. Spurlino, and L.E. Rodseth, *Extensive features of tight oligosaccharide binding revealed in high-resolution structures of the maltodextrin transport/chemosensory receptor.* Structure, 1997. **5**(8): p. 997-1015.
